# Should Exercise Be Prescribed Differently Between Women and Men? An Emphasis on Women Diagnosed With Parkinson's Disease

**DOI:** 10.3389/fphys.2018.01040

**Published:** 2018-08-02

**Authors:** Brandon R. Rigby, Ronald W. Davis

**Affiliations:** Department of Kinesiology, Texas Woman's University, Denton, TX, United States

**Keywords:** aerobic, female, flexibility, functional abilities, neurodegenerative, resistance

## Introduction

Parkinson's disease (PD) is a neurodegenerative disease caused by a reduction in dopaminergic neurons in the substantia nigra (Dexter and Jenner, 2013). Individuals can exhibit muscle tremors, rigidity, and bradykinesia, leading to posture and gait abnormalities (Dexter and Jenner, 2013). Although the incidence ratio of males to females is ~1.46 (Taylor et al., 2007), the prevalence of PD among men is doubled when compared to women (Elbaz et al., 2002). A lower mortality rate has traditionally been associated with PD in women due to the overall longer life expectancy when compared to men in the general population (Xu et al., 2014; Pinter et al., 2015). However, a diagnosis of PD was found to be associated with a two-fold increased risk for all-cause mortality in a recent study with 396 older women (Winter et al., 2016). This value is similar to that reported among older men (Xu et al., 2014).

Males may have a greater predisposition to develop PD (Gillies et al., 2014). This bias may be due to the molecular pathology of PD. Gene expression profiles in dopaminergic neurons are sex-specific, and the survivability of these neurons are dependent on molecular pathways that are very different in men and women (Gillies et al., 2014). In healthy brain tissue, genes involved in signal transduction and neuronal growth are up-regulated more in women (Cantuti-Castelvetri et al., 2007). In men, genes with specific mutations (e.g., α-synuclein, PINK-1) that may contribute to the pathogenesis of PD are upregulated more often (Simunovic et al., 2010). A downregulation of genes that provide for oxidative phosphorylation, and synaptic and nerve impulse transmission, is also more prevalent in older males (Simunovic et al., 2010). Therefore, women are thought to have greater protection from PD when compared to men, which may also be due to estrogen concentrations (Gillies et al., 2014).

Estrogen influences dopamine synthesis and release while inhibiting dopamine uptake (Shulman, 2007). The higher concentrations of estrogen are a possible reason for the more benign phenotype in women when compared to men, particularly before a course of medication has begun (Haaxma et al., 2007; Miller and Cronin-Golomb, 2011; Cereda et al., 2013). Estrogen may play a role in preventing toxins from possibly degrading neurons in the substantia nigra (Shulman, 2007). Exogenous or endogenous estrogen administration may therefore play a crucial role in the pathogenesis of PD (Lv et al., 2017). There are several factors, including medicinal options, surgical interventions, dietary strategies, and lifestyle habits that may alter estrogen levels in women with PD. Oral contraceptive (OC) use may increase the risk of PD among women (Nicoletti et al., 2011), with a 20% increased risk of developing PD for every five years of OC use (Simon et al., 2009). However, the use of OCs has been also been shown to be inversely associated with PD risk (Greene et al., 2014), with continuous use for more than 10 years determined to even be a protective mechanism against developing PD (Liu et al., 2014). Following an ovariectomy, a decrease in the concentration of estrogen receptor-α and an increase in angiotensin, NADPH-oxidase activity, and the expression of neuroinflammatory markers was observed in the substantia nigra of menopausal rats (Rodriguez-Perez et al., 2015). Smoking may increase levels of estrogen and serum sex hormone-binding globulin, and may also be protective against PD, as an inverse correlation between smoking and PD risk has been shown (Ritz et al., 2007; Breckenridge et al., 2016). Proper intake of vitamin D may increase glial derived neurotrophic factor (Smith et al., 2006) and reduce activated microglial cells (Kim et al., 2006), therefore acting as a neuroprotectant with estrogen and reducing inflammation. The protective effect of estrogen is not always evident, particularly in women who report a more rapid onset of motor symptoms upon diagnosis (Sato et al., 2006; Colombo et al., 2015; Bjornestad et al., 2016).

With regard to motor symptoms, women typically present more with tremors and bradykinesia, but not rigidity, when compared to men (Haaxma et al., 2007; Martinez-Martin et al., 2012). Non-motor symptoms, such as constipation, restless legs, pain, nervousness, anxiety, and sadness, are more prevalent in women (Martinez-Martin et al., 2012; Solla et al., 2012; Picillo et al., 2013; Szewczyk-Krolikowski et al., 2014). A reduction in visuospatial cognition also occurs more frequently in women (Miller and Cronin-Golomb, 2011).

To treat the motor and non-motor symptoms of PD, individuals are often presented with a variety of options. Medications and surgical procedures are available but are often unsuccessful at treating all symptoms of PD, can be expensive and invasive, and may lead to unwanted side effects (Bloem et al., 2004). Exercise may therefore be an inexpensive and complementary option to other interventions to treat symptoms of PD. Performing regular exercise may improve numerous functional outcome measures and quality-of-life, particularly in higher-functioning individuals diagnosed with neurodegenerative diseases, including PD, multiple sclerosis, and dementia (Dodd et al., 2011; Brienesse and Emerson, 2013; Morley et al., 2015).

## Exercise and Parkinson's disease

Gait speed is slower in older women, possibly due to observed increases in muscle strength and standing balance ability in men (Bohannon, 1997). When compared to men with PD, women with PD exhibit increased cadence and decreased stride length and frequency (Kokko et al., 1997; Pedersen et al., 1997). To improve gait, motor performance, and quality-of-life, aerobic exercise on a motorized treadmill may be effective intervention for those with PD (Herman et al., 2007). Treadmill exercise can improve motor function, stride and swing time variability, and gait speed in adults with mild to moderate PD (Herman et al., 2007). This in turn may allow for greater mobility during activities of daily living (ADLs) and a reduced incidence of falls (Cakit et al., 2007; Herman et al., 2007). Performance during ADLs may be enhanced more with the use of external sensory cues during exercise (Cassimatis et al., 2016). These auditory, visual, or tactile cues bypass the basal ganglia using alternative pathways (e.g., cortical, parietopremotor) when processed, thus creating an additive improvement in ADL performance (Kones, 2010).

Resistance and flexibility exercises are also recommended for individuals diagnosed with PD. Resistance exercise training can improve muscle strength, muscular endurance, fat-free mass, balance and mobility, and walking economy in adults with PD (Brienesse and Emerson, 2013). When assessing lower limb strength, Pääsuke et al. (2002) found that reaction times and maximum isometric force values were different between legs in women with PD. This may be due to the postural and balance impairments that are prevalent with a diagnosis of PD. Using regression analysis, Schenkman et al. (1998) found a greater correlation between women with PD and poor spinal flexibility and balance when compared to men with PD.

Alternative modalities of exercise, including Nordic walking (Cugusi et al., 2017), tai chi (Hackney and Earhart, 2008), and tango dancing (Hackney et al., 2007), may also elicit improvements in balance and mobility in those with PD. Virtual reality (VR) is a newer technology that may be integrated into a rehabilitative program for those with PD. By allowing for repetitive movement that targets both motor and cognitive performance, VR may allow for the learning or re-learning of motor strategies that have been lost due to the progression of PD (Goble et al., 2014).

## Exercise prescription recommendations

Traditional aerobic and resistance exercise should be prescribed differently to women diagnosed with PD due to the possible inherent sex differences in this population. Current exercise guidelines for adults with PD and proposed guidelines for women with PD are found in Table 1. As PD is a risk factor for osteoporosis, appropriate aerobic modalities should be chosen for women with PD. Short, supervised walks on a level surface with occasional turns (e.g., an athletic track), to add variation to the direction of loads, may be the best aerobic exercise modality that can be prescribed to women with PD. If not ambulatory, leg ergometry may be a suitable option, with a focus on resistance rather than speed. Intensity should be prescribed using VO_2_ reserve (if available) or rate of perceived exertion (RPE). Prescribing intensity using heart rate responses should not be recommended, as many individuals with PD exhibit autonomic nervous system (ANS) dysfunction (e.g., sympathetic denervation) and are prescribed medications that can interfere with heart rate responses and heart rate variability (Ziemssen and Reichmann, 2010; Moore et al., 2016). This ANS dysfunction is thought to contribute to fatigue in those with PD (Nakamura et al., 2011). Session duration and frequency, particularly at the start of an exercise program, should therefore be limited.

**Table 1:** Exercise guidelines for adults diagnosed with Parkinson's disease.

**Modality**	**Frequency**	**Intensity**	**Duration**
**CURRENT GUIDELINES FOR ADULTS WITH PD**			
**Aerobic**			
Leg and arm ergometry Walking Cycling	3–4 days/week	30–60% VO_2_R 30–60% HRR 55–75% HR_max_ RPE 9–13	30–60 min/session Up to 6 sessions/day (for walking only)
**Resistance**			
Machines and free weights Closed-chain kinetic exercises	3–4 days/week	40–80% 1RM	1–3 sets of 8–12 reps
**Flexibility**			
Static stretching	3 days/week	Joints at full flexion/extension	Up to 30 sec/stretch
**PROPOSED GUIDELINES FOR WOMEN WITH PD**			
**Aerobic**			
Multi-directional walking (if ambulatory) Leg ergometry (if not ambulatory)	Begin: 1–2 days/week Progress to: 4+ days/week	30–60% VO_2_R RPE 9–13	30–60 min/session Up to 6 sessions/day (for walking)
**Resistance**			
Squats Closed-chain kinetic exercises Machines	Begin: 1 day/week Progress to: 2-3 days/week	Against body weight, up to 80% 1RM	1–3 sets of 8–12 reps
**Flexibility**			
Static and dynamic stretching	3–5 days/week	Joints at full flexion/extension	Up to 30 sec/stretch

*Current guidelines adapted from Moore et al. (2016) and Jacobs (2018). Establishing a conservative 5RM or 10RM may be needed for some clients. VO_2_R = VO_2_ reserve; HR_max_ = maximum heart rate; HRR = heart rate reserve; RPE = rate of perceived exertion; 1RM = 1 repetition maximum*.

Squats using only body weight or with additional weight may be the most appropriate resistance exercise modality for women with PD. Squats are closed-chain kinetic exercises, which may be a more suitable exercise due to the abnormal coordination between limbs caused by a greater prevalence of tremors at rest in women with PD (Ehrman et al., 2013). The strength of the quadriceps, the prime movers during the upward phase of a squat, is correlated with functional abilities and performance during ADLs (Moore et al., 2016). Targeting the quadriceps musculature during exercise is beneficial because the extensors typically exhibit more muscle weakness than the flexors, and there is evidence that proximal muscles are weaker than distal muscles in those with PD (Inkster et al., 2003; Cano-de-la-Cuerda et al., 2010). The induced compression during the exercise may also improve bone mineral density (BMD) at the hip. Machine weights may be an added supplement to resistance training once increases in volume and frequency can be tolerated.

Flexibility training needs to be incorporated into an exercise program for those with PD. A combination of stretching and resistance exercise can improve muscle strength and gait speed in this population (Shulman et al., 2013). As women with PD typically exhibit more frequent episodes of bradykinesia, increasing the strength of skeletal muscles (particularly lower body) and gait parameters is crucial (Haaxma et al., 2007).

## Conclusions

The Office of Research on Women's Health (ORWH) within the National Institutes of Health (NIH) has called for research scientists to consider sex when designing study protocols to allow for both men and women to receive the full benefit of medical research (National Institutes of Health, 2017). Exercise is an effective intervention that can elicit functional changes in adults with PD. Women with PD should follow an exercise prescription that somewhat deviates from the current exercise guidelines, which are not categorized according to sex. This deviation is due in part to the differences observed with the timing of PD onset and progression, primary symptoms, and hormonal changes that are exhibited in women with PD. A comparison of the physiological effects of exercise training between men and women with PD needs to be further investigated to allow clinicians and health practitioners to correctly prescribe exercise as a long-term option to treat the symptoms of PD.

## Author contributions

All authors listed have made a substantial, direct and intellectual contribution to the work, and approved it for publication.

## Conflict of interest statement

The authors declare that the research was conducted in the absence of any commercial or financial relationships that could be construed as a potential conflict of interest.
